# Effect of cisternostomy in managing cerebral swelling: A clinical study

**DOI:** 10.6026/973206300223551

**Published:** 2026-06-30

**Authors:** Kumar Abhinav, Abhishek Chaudhary, Rajiv Rajan

**Affiliations:** 1 Department of Neurosurgery, Patna Medical College & Hospital, Patna, Bihar, India

**Keywords:** Cerebral edema, cisternostomy, decompressive craniectomy, glasgow outcome scale (GOS), intracranial pressure (ICP)

## Abstract

Traumatic brain injury (TBI) often leads to cerebral edema, resulting in elevated intracranial pressure (ICP) and poor neurological outcomes. Therefore, it is of interest to evaluate the efficacy of cisternostomy in managing cerebral edema, showing a significant reduction in ICP and improved neurological outcomes, including Glasgow Coma Scale (GCS) and Glasgow Outcome Scale (GOS) scores. The procedure offers a less invasive alternative to decompressive craniectomy with fewer complications and favorable recovery. Data from 200 patients suggest its potential for improving long-term functional recovery. Thus, we report cisternostomy role as a viable intervention for ICP reduction and functional recovery in TBI patients.

## Background:

Cerebral edema, or brain swelling, is a critical condition that often results from traumatic brain injury (TBI), ischemic stroke, or various other pathologies affecting the brain [1]. It leads to an increase in intracranial pressure (ICP), which can disrupt normal cerebral perfusion, impair brain function and ultimately threaten life if left untreated. The management of cerebral edema remains one of the most challenging aspects of neurocritical care, as the delicate balance between reducing brain swelling and maintaining sufficient blood flow to the brain is paramount [2]. One potential surgical intervention that has gained attention in recent years is cisternostomy, a procedure aimed at alleviating elevated intracranial pressure through the selective drainage of cerebrospinal fluid (CSF) from the basal cisterns. This procedure's efficacy, however, remains a subject of ongoing research and debate [3]. Cisternostomy involves the surgical creation of a drainage pathway that allows the controlled release of CSF from the cisterns located at the base of the brain. These cisterns, which house the cerebrospinal fluid surrounding the brainstem and cranial nerves, are crucial to maintaining the balance of pressure within the cranial cavity [4]. In cases of cerebral edema, this drainage can reduce ICP by decreasing the volume of CSF, thereby preventing further neurological damage caused by elevated pressure. Unlike conventional treatments such as osmotherapy and decompressive craniectomy, cisternostomy is considered a minimally invasive procedure that has the potential to offer a more controlled, precise approach to managing brain swelling without the need for extensive surgery [5]. The importance of investigating the efficacy of cisternostomy arises from the fact that current treatment modalities for cerebral edema often come with significant risks and limitations. Osmotic agents, such as mannitol and hypertonic saline, are commonly used to reduce intracranial pressure, but they may cause electrolyte imbalances, renal failure and hemodynamic instability [6]. Decompressive craniectomy, a more invasive surgical approach, can effectively reduce ICP but at the cost of exposing the brain to potential infections, delayed cerebral atrophy and other complications. Therefore, an alternative procedure that offers a less invasive yet effective solution is a critical area of research [7]. Previous studies have explored the use of cistern ostomy in various clinical settings, with some showing promising results in reducing ICP and improving patient outcomes, while others suggest mixed or inconclusive benefits. The variability in outcomes may stem from differences in patient populations, surgical techniques and the timing of intervention [8]. As such, a comprehensive study investigating the efficacy of cistern ostomy in patients with cerebral edema is essential to better understand its potential advantages and limitations compared to other established treatment methods [9]. Therefore, it is of interest to determine the potential of cistern ostomy as an effective, minimally invasive treatment for cerebral edema, offering insights into its role in improving patient outcomes, reducing intracranial pressure and enhancing recovery in neurocritical care.

## Methodology:

## Study design:

This study employed a prospective, observational cohort design to evaluate the efficacy of cistern ostomy in patients with cerebral edema. The research was conducted at a tertiary care hospital with a specialized neurocritical care unit. The study involved a sample of 200 participants who met the inclusion criteria, with follow-up evaluations conducted over a six-month period to assess long-term outcomes.

## Inclusion criteria:

The study included adult patients (aged 18-65) diagnosed with significant cerebral edema secondary to traumatic brain injury (TBI), ischemic stroke, or other neurological conditions that resulted in increased intracranial pressure. Participants had elevated ICP despite conservative management and cistern ostomy was deemed a suitable intervention by the treating neurosurgical team. Patients who had contraindications to surgery, such as severe coagulopathy, active infections, or severe underlying comorbidities, were excluded from the study.

## Sample size calculation:

A sample size of 200 participants was selected to ensure sufficient statistical power to detect meaningful differences in primary and secondary outcomes. The sample size was calculated using the formula for comparing two independent proportions with a power of 80% and a significance level of 0.05. Based on previous studies and expected effect sizes, a sample size of 200 participants provided enough power to assess the impact of cistern ostomy on intracranial pressure reduction, neurological outcomes and patient survival.

## Data collection:

Data were collected from the medical records of patients who met the inclusion criteria and consented to participate in the study. The following data points were collected at baseline, immediately post-surgery and during the follow-up period:

[1] Demographic Information: Age, gender, medical history and baseline health status.

[2] Clinical Parameters: Glasgow Coma Scale (GCS) score at admission, initial ICP measurements and cause of cerebral edema (*e.g.*, TBI, stroke).

[3] Surgical Procedure: Details of the cistern ostomy procedure, including the timing of the intervention, technique used and intraoperative complications.

[4] Intracranial Pressure (ICP) Measurements: Pre- and post-operative ICP levels, monitored continuously for 72 hours after surgery.

[5] Neurological Outcomes: GCS score, neurological examinations and the extent of recovery at 1 month, 3 months and 6 months post- procedure.

[6] Mortality and Morbidity Rates: Patient survival rates and any post-operative complications (*e.g.*, infection, bleeding, reoperation).

[7] Other Outcomes: Length of ICU stay, time to neurological recovery and discharge disposition (*e.g.*, home, rehabilitation, long-term care).

## Surgical intervention:

Cistern ostomy was performed by experienced neurosurgeons, with the procedure tailored to each patient's specific condition. The surgical technique involved the creation of a drainage pathway for cerebrospinal fluid from the basal cisterns and post-operative ICP monitoring was implemented to evaluate the immediate impact of the procedure on ICP reduction.

## Follow-up and outcome assessment:

Patients were followed up for six months' post-surgery. Outcome measures were assessed at the following time points:

[1] Pre-operative: Baseline ICP, GCS score and clinical assessment.

[2] Postoperative: Immediate post-surgical ICP measurements, GCS score and complications.

[3] 1 Month: Neurological examination, GCS score and functional recovery.

[4] 3 Months: Neurological status, functional outcomes and cognitive assessment.

[5] 6 Months: Long-term survival, cognitive and motor recovery and quality of life (using standard outcome scales such as the Glasgow Outcome Scale and Functional Independence Measure).

## Statistical analysis:

Data analysis was performed using SPSS (version 25.0) or a similar statistical software package. The primary outcome, reduction in ICP, was analysed using paired t-tests to compare pre- and post-operative ICP levels. Secondary outcomes, including neurological recovery, survival and complications, were analysed using chi-square tests for categorical variables and independent t-tests or ANOVA for continuous variables. Kaplan-Meier survival curves were used to evaluate survival rates over time. Multivariate regression analysis was performed to identify factors that predicted successful outcomes following cistern ostomy.

## Results:

The present study included 200 patients who underwent cistern ostomy for the management of cerebral edema. The baseline demographic and clinical characteristics of the study participants are summarized in Table 1. The mean age of the patients was 45.7 ± 12.3 years, with males constituting 60% of the study population and females accounting for 40%. Traumatic brain injury was identified as the most common cause of cerebral edema (50%), followed by ischemic stroke (35%) and other causes such as brain tumors (15%) (Table 1). The primary outcome of the study was the reduction in intracranial pressure (ICP) following the cistern ostomy procedure. As presented in Table 2, there was a significant reduction in ICP from preoperative to postoperative measurements. The mean preoperative ICP was 27.5 ± 5.2 mmHg, which decreased to 15.6 ± 3.3 mmHg following surgery. The mean reduction in ICP was 11.9 mmHg and this difference was found to be statistically significant (p < 0.001) (Table 2). Neurological recovery was assessed using the Glasgow Coma Scale (GCS) at different postoperative intervals. The progression of GCS scores over time is shown in Table 3. The mean preoperative GCS score was 6.4 ± 2.3, indicating severe neurological impairment at presentation. At 1 month postoperatively, the mean GCS score improved significantly to 10.3 ± 1.9. Further improvement was observed at 3 months, with a mean GCS score of 13.1 ± 1.1, and by 6 months, the average score increased to 14.2 ± 0.7. The improvement in GCS scores across all follow-up intervals was statistically significant (p < 0.001) (Table 3). Mortality and postoperative morbidity outcomes are summarized in Table 4. Out of the total 200 patients, 18 patients (9%) died within six months following the procedure. Most deaths occurred within the first postoperative month. Infections were reported in 12 patients (6%), postoperative bleeding occurred in 7 patients (3.5%), and reoperation was required in 5 patients (2.5%). Although complications were encountered, they were managed conservatively and no significant morbidity was directly attributed to the cistern ostomy procedure (Table 4). The average length of stay in the intensive care unit (ICU) varied according to the underlying etiology of cerebral edema and is presented in Table 5. Patients with traumatic brain injury had the longest ICU stay, with a mean duration of 9.1 ± 4.2 days. Patients with ischemic stroke had a comparatively shorter ICU stay of 5.4 ± 2.3 days, while patients with other causes of cerebral edema had a mean ICU stay of 6.3 ± 3.5 days. The variation in ICU stay among the different conditions was statistically significant (p < 0.001) (Table 5). Functional recovery was evaluated using the Glasgow Outcome Scale (GOS) over a six-month follow-up period. As shown in Table 6, favourable outcomes (GOS score 4-5) were observed in 120 patients (60%) at 1 month postoperatively. This number increased to 150 patients (75%) at 3 months and further improved to 155 patients (77.5%) at 6 months. These findings indicate progressive functional recovery and positive neurological outcomes in a substantial proportion of patients following cistern ostomy (Table 6).

## Discussion:

This study evaluated the efficacy of cisternostomy as a treatment for cerebral edema, particularly in the context of traumatic brain injury (TBI). The findings align with several key studies that have explored cisternostomy's role in ICP management, neurological recovery and functional outcomes. When compared to compressive craniectomy (DC), cisternostomy is shown to be a less invasive yet effective intervention in reducing ICP and improving patient outcomes. Chandra *et al.* (2020) [10] highlighted cisternostomy as a promising alternative to decompressive craniectomy for TBI patients, reporting a significant reduction in ICP and improvement in Glasgow Outcome Scale (GOS) scores. Their findings were similar to those of our study, where we observed a marked decrease in ICP following cisternostomy. In addition, Kumarasamy *et al.* (2021) [11] further reinforced the efficacy of cisternostomy, specifically its ability to control ICP in severe TBI cases. Their study suggests that cisternostomy could be utilized as either an adjuvant or standalone intervention, with significant ICP reduction and improved functional outcomes. Similar to Kumarasamy *et al.* our study showed that cisternostomy led to a substantial reduction in ICP, as well as notable improvements in GCS and GOS scores. The versatility of cisternostomy, as demonstrated in their work, was also apparent in our findings, where patients with varying etiologies of cerebral edema responded well to the intervention. Eraky *et al.* (2022) [12] provided a more case-based approach, emphasizing the theoretical mechanisms behind cisternostomy's effect on prolonged ICP elevation. They found that the procedure effectively reduced ICP, particularly in cases where conventional methods like osmotherapy were insufficient. This aligns with our study, where cisternostomy was shown to be an effective alternative when traditional management failed to achieve desired ICP reduction. Eraky *et al.'s* [12] exploration of prolonged ICP elevation mirrors our findings, where cisternostomy played a pivotal role in cases where rapid intervention was needed, especially in patients who had experienced significant neurological damage. Giammattei *et al.* (2019) [13] further contributed to the evidence supporting cisternostomy by reporting on outcomes in patients with severe head injuries. Their cohort study found that cisternostomy resulted in positive GOS improvements and reduced the need for reoperation. Their results are consistent with our findings, as we observed a similar trend of favorable recovery in patients, particularly in those with moderate to severe TBI. The ability of cisternostomy to decrease ICP and improve GOS scores in these patients highlights its potential as a standard procedure in the neurocritical care setting. Ramirez *et al.* (2021) [14] conducted a multi-center study examining the role of cisternostomy in TBI management and found favorable outcomes, particularly when combined with decompressive craniectomy. However, they also noted that cisternostomy could be an effective standalone approach, especially for patients who were not candidates for decompressive craniectomy due to contraindications. Their findings align with our study's results, where cisternostomy was used independently to manage elevated ICP, leading to similar outcomes to those achieved with more invasive procedures. Finally, Satyarsa *et al.* (2023) [15] conducted a systematic review and meta-analysis that compared cisternostomy and decompressive craniectomy across various clinical studies. The meta-analysis concluded that while decompressive craniectomy offers rapid ICP reduction, cisternostomy demonstrated comparable efficacy with fewer complications, making it a preferred option for many patients. Our study agrees with this conclusion, as it supports the idea that cisternostomy, with its less invasive nature, can achieve similar results to decompressive craniectomy without the associated long-term risks.

## Conclusion:

Cisternostomy proves to be an effective and minimally invasive procedure for reducing intracranial pressure and improving neurological outcomes in patients with cerebral edema. Thus, along with supporting evidence from previous research, highlight its potential as a preferred alternative to decompressive craniectomy. Further investigations are needed to refine patient selection and optimize its clinical application in neurocritical care.

## Figures and Tables

**Table 1:** baseline demographic and clinical characteristics of study participants

**Characteristic**	**Value**
Total Sample Size	200
Mean Age (years)	45.7 (±12.3)
Gender Distribution	
- Male	120 (60%)
- Female	80 (40%)
Cause of Cerebral Edema	
- Traumatic Brain Injury	100 (50%)
- Ischemic Stroke	70 (35%)
- Other (e.g., brain tumors)	30 (15%)

**Table 2:** Preoperative and postoperative ICP Measurements

**Time Point**	**ICP (mmHg)**	**Mean Difference (p-value)**
Preoperative	27.5 (±5.2)	-
Postoperative	15.6 (±3.3)	11.9 mmHg (p <0.001)

**Table 3:** Improvement in glasgow coma scale (GCS) Score over time

**Time Point**	**GCS Score (Mean ± SD)**	**p-value**
Preoperative	6.4 (±2.3)	-
1 Month	10.3 (±1.9)	p < 0.001
3 Months	13.1 (±1.1)	p < 0.001
6 Months	14.2 (±0.7)	p < 0.001

**Table 4:** Mortality and morbidity rates

**Outcome**	**Number of Patients**	**Percentage (%)**
Mortality	18	9
Infection	12	6
Bleeding	7	3.5
Reoperation	5	2.5

**Table 5:** Length of ICU Stay

**Condition**	**ICU Stay (Mean ± SD)**	**p-value**
Traumatic Brain Injury	9.1 (±4.2)	p < 0.001
Ischemic Stroke	5.4 (±2.3)	
Other Causes of Cerebral Edema	6.3 (±3.5)	

**Table 6:** Glasgow outcome scale (GOS) Recovery over time

**Time Point**	**Favorable Outcome (GOS 4-5)**	**Percentage (%)**
1 Month	120	60
3 Months	150	75
